# Case report: Tremor in the placebo condition of a blinded clinical trial of intermittent theta-burst stimulation for cocaine use disorder

**DOI:** 10.3389/fpsyt.2024.1391771

**Published:** 2024-07-09

**Authors:** Vaughn R. Steele, Alexander Rotenberg, Noah S. Philip, Mark Hallett, Elliot A. Stein, Betty Jo Salmeron

**Affiliations:** ^1^ Department of Psychiatry, Yale University School of Medicine, New Haven, CT, United States; ^2^ Olin Neuropsychiatry Research Center, The Institute of Living at Hartford Hospital, Hartford, CT, United States; ^3^ Center for Brain and Mind Health, Yale University, New Haven, CT, United States; ^4^ Department of Neurology, Boston Children’s Hospital, Harvard Medical School, Boston, MA, United States; ^5^ Department of Psychiatry and Human Behavior, The Warren Alpert Medical School, Brown University, Providence, RI, United States; ^6^ Center for Neurorestoration and Neurotechnology, VA Providence Healthcare System & Department of Psychiatry and Human Behavior, Alpert Medical School of Brown University, Providence, RI, United States; ^7^ National Institute of Neurological Disorders and Stroke, Intramural Research Program, National Institutes of Health, Bethesda MD, United States; ^8^ Neuroimaging Research Branch, National Institute on Drug Abuse, Intramural Research Program, National Institutes of Health, Baltimore, MD, United States

**Keywords:** iTBS, cocaine use disorder, placebo effect, low-intensity rTMS, sham rTMS

## Abstract

We report a case of a new-onset, persistent tremor that developed during a clinical trial (NCT02927236) of intermittent theta burst stimulation [iTBS, a form of repetitive magnetic transcranial magnetic stimulation (rTMS)] for cocaine use disorder. Although the participant exhibited an exceptionally strong clinical response, subsequent unblinding revealed that they received sham iTBS. This case highlights the potential for strong functional neurological placebo responses in rTMS trials, and functional disorders might be a marker of a placebo response. Additionally, we note the possibility that the weak e-fields produced by some sham rTMS systems may induce clinically relevant effects.

## Introduction

Transcranial magnetic stimulation (TMS) has a demonstrated capability to generate significant placebo responses (1, 2), as would be expected given the complexity of the treatment setting, significant somatic stimulation, and novelty of the intervention (3). In addition to this potential for inducing placebo responses, growing literature suggests that low-intensity electromagnetic field exposure such as that produced by widely used sham rTMS systems may induce biological effects (4, 5). Given these two considerations, we present a case of new-onset, persistent functional tremor in a participant who experienced a strong clinical response to an intermittent theta-burst stimulation (iTBS) intervention for cocaine use disorder (CUD) during a randomized, sham-controlled, double-blind clinical trial (RCT; NCT02927236).

## Case description

**Figure 1:**
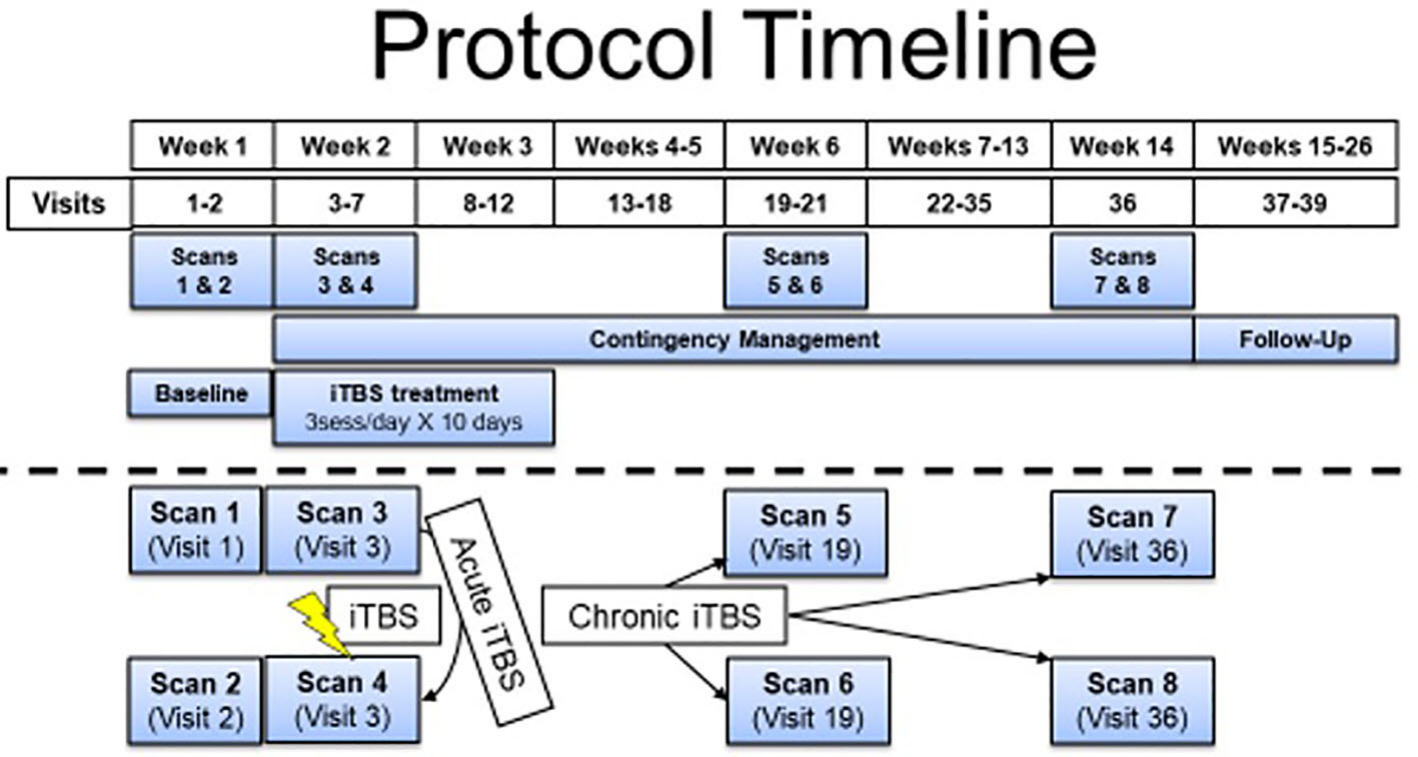
Protocol timeline separated by weeks. The participant described in this report received 5 days of iTBS before discontinuing the intervention and moving to the follow-up phase. He received contingency management as the treatment as usual throughout his participation (starting when iTBS was initiated). fMRI measures were acquired prior to iTBS initiation and before and after the iTBS sessions on the first day of iTBS. No additional experimental fMRI scans were completed with this participant because he was lost to follow-up.

The participant was a 47-year-old TMS-naïve, Caucasian male with severe CUD who provided written informed consent to this National Institute on Drug Abuse (NIDA) protocol approved by the NIDA Institutional Review Board (IRB) and Food and Drug Administration (FDA). The participant had hoped to participate in the pilot arm of this study (6), which concluded before he could enroll. He waited approximately one year while we obtained FDA clearance to proceed with the RCT (see **Figure 1** for the study timeline). He continued to struggle with CUD during this year and was eager to participate in the double-blind study, with a strong expectation of benefit. Screening evaluation was largely unremarkable and included history and physical exam, complete blood cell count, basic chemistries, liver function tests, thyroid stimulating hormone, erythrocyte sedimentation rate, HIV, syphilis and hepatitis B/C screening, urine toxicology for drugs of abuse and therapeutic medications, electrocardiogram, diagnostic, and statistical Manual of Mental Disorders (DSM-5), mini-International Neuropsychiatric Interview (MINI), with follow-up clinical interview, DSM-5 substance use disorders evaluation, and TMS and magnetic resonance imaging (MRI) safety screening.

The participant had used cocaine for about 24 years, daily for about 16 years, and met the criteria for severe CUD. Approximately 5 years prior to study entry, his usage escalated from about $30 daily to about $80 daily, mostly crack but occasionally snorting powdered cocaine. He also smoked 10–20 cigarettes per day for 30 years and one cannabis blunt daily for about 20 years, although he did not meet the criteria for a cannabis use disorder diagnosis. He occasionally (monthly or less) used oxycodone when offered at parties. He denied using benzodiazepines but presented with positive urine tests for benzodiazepines on several occasions prior to study entry, which may have been related to taking what he was told was Methyl enedioxy methamphetamine (MDMA) at a party. However, some of these initial positive urine results failed to be confirmed via positive mass spectrometry and thus likely represented urine tests that were false positives. He admitted occasional MDMA use when offered at parties and was urine positive once during the year in which he was involved in studies at NIDA. He tried to quit using cocaine about 15 times, with one episode of formal treatment, in the 5 years prior to study entry; however, no significant periods of abstinence followed any quit attempt. He accepted our suggestion to seek a therapist to help manage various family issues, relationship difficulties, and mild depressive symptoms. He continued in therapy off and on while he waited for our study to begin, but he declined a specific referral for treatment of his CUD. He had no history of head injuries, seizures, or any other neurological problems.

The rTMS RCT treatment-imaging research study was designed to test iTBS as an adjuvant to contingency management treatment for CUD. During this trial, repeated functional magnetic resonance imaging (fMRI) scans are conducted to elucidate changes in neural functioning due to the iTBS intervention (see **Figure 1** for study timeline). The study delivers accelerated iTBS to left dorsolateral prefrontal cortex [MNI coordinates: −50, 30, and 36 (7)] using Brainsight for targeting guidance at 100% resting motor threshold (RMT), determined using the PEST software (8) (adjusted for difference in distance from scalp to cortex at motor hotspot versus dorsolateral prefrontal cortex). RMT was determined by the motor hotspot, defined as the region of the left motor cortex that reliably elicited movement of the contralateral first dorsal interosseous (FDI) muscle and/or an associated motor-evoked potential (MEP). TMS stimulation that elicited any movement in the contralateral hand and/or a MEP of at least 50 µV was counted as a positive response when determining RMT.

All TMS was applied using a MagVenture MagPro 100 with MagOption and a figure-8 Cool-B65 Active/Placebo coil (MagVenture Inc., Alpharetta, GA, USA). We applied the standard iTBS sequence, 600 biphasic pulses delivered over 190 s (9), in an accelerated fashion by administering three sessions each day with intersession intervals of at least 60 min. During iTBS, participants view cocaine-related pictures displayed on a computer monitor and are encouraged to actively resist craving during iTBS sessions; CBT-based intervention training on resistance strategies is provided prior to the session. Engaging the targeted circuit has the potential for greater treatment efficacy (10). Ten treatment days for accelerated iTBS are scheduled within a 3-week period. Our sham procedure delivers electrical stimulation via Magventure scalp electrodes placed 3 cm apart on the participant’s forehead near the hairline and as close to the iTBS target as possible. Based on internal testing prior to the start of the protocol, we set the intensity to nine out of 10, which produced sensory characteristics similar in character and intensity to the active iTBS stimulation. These rTMS procedures are identical to those in our pilot study (6), which included our standard ramping procedures. Ramping of the stimulator output started about 20 percentage points below RMT, which allowed a gradual increase of intensity as tolerated by the participant. Participants affirmed ramping between trains until they received two trains at their RMT, which was deemed a successful toleration. If the iTBS administration was too painful (i.e., intolerable), participants could cease administration at any point. Generally, the stimulator was ramped by 5 points between each iTBS train until reaching RMT. Each iTBS session was ramped up with these procedures. The TMS operators (VRS and BJS) received training on all TMS procedures through the TMS Intensive Course at the Medical University of South Carolina; each had three years of experience administering the protocol.

In the week prior to the treatment phase of the study, the participant completed a characterization phase, which consisted of two MRI scans, multiple questionnaires, and bench assessments, as well as an iTBS toleration session to familiarize him with the study procedures and the sensations of the iTBS condition to which he was randomly assigned (week 1 of **Figure 1**). He was admitted to an inpatient unit on a Monday evening with the expectation of study onset the following morning. However, the start of his treatment was delayed after he tested positive for benzodiazepines as well as cocaine (American Screening Corporation, Shreveport, LA, USA, urine test, which screens for buprenorphine, benzodiazepine, cocaine, amphetamine, methamphetamine, MDMA, morphine, methadone, oxycodone, PCP, and THC). He denied any use, and as the subsequent confirmatory mass spectrometry did not reveal benzodiazepines in his urine, he began treatment on the following Thursday.

RMT was determined each day and varied between 50% and 53% of the maximum stimulator output (MSO). He received two full days of treatment (six total iTBS sessions) on Thursday and Friday. He found the treatments very uncomfortable, consistent with all previous pilot study participants, but was able to tolerate them and wished to continue in the study. He was discharged over the weekend and intended to avoid using cocaine by spending time with his family. However, that plan was unsuccessful, as he reported buying $10 of cocaine on Friday night upon discharge from the inpatient unit. Upon his return to the research unit on Sunday night, he reported that he did not get his usual high following cocaine use and decided not to use all the cocaine he purchased, which was very unusual for him. These reported effects are similar to participants’ reports in our pilot, where only active iTBS was applied (6). He tested positive for cocaine and benzodiazepines and again denied benzodiazepine use.

He resumed iTBS treatments Monday morning (day 3), after testing positive for cocaine but negative for benzodiazepines. On Tuesday morning (day 4), he reported that his usual nicotine cigarettes tasted stale and that he had reduced his smoking to five cigarettes per day (down from 10 to 20), noting a markedly reduced craving for cigarettes. On Wednesday (day 5), the participant, who regularly experienced vivid “drug dreams” prior to study entry, reported having had dreams of the same vivid quality but about food and gardens, something he had never experienced before. He reported 8–10 h of sleep the night before each study day (day 1: 8 h; day 2: 9 h; day 3: 8 h; day 4: 10 h, day 5: 8 h). His RMT was 53% MSO, adjusted to account for the scalp-to-cortex distance to 48% delivered over left DLPFC. Toward the end of his second iTBS session that day, he exhibited some movement in his right hand (approximately 2 Hz supination/pronation at the wrist) coincident with the 2-s “on” period of iTBS. The movements appeared related to tensing to withstand the discomfort associated with iTBS delivery. He verbally affirmed his well-being, and the last few trains were then completed. Following session completion, he once again displayed intermittent tremors in the right hand, much like what had occurred during the treatment session, which stopped when he clenched the hand into a fist. He also developed a headache (5/10 pain) after this session and received acetaminophen, 650 mg orally. The third session was delayed for about 20 min (such that the interval was 80 min instead of the planned 60 min) due to the headache. He was headache- and tremor-free before the start of the third session that day, which was completed without incident. Other than headaches, no other adverse events were reported. After receiving all iTBS sessions on day 1, he reported a headache (2/10 pain) lasting about 30–45 min and resolving spontaneously. On day 3, he reported a headache (3–4/10 pain), for which he first declined medication but then took acetaminophen, and it resolved in about 30 min. On day 5, the day the tremor began, he reported a headache (5/10 pain) between the second and third treatments that responded to acetaminophen (described above).

After returning to the nurses’ station (< 5 min), however, his hand once again began shaking, and he reported a return of his headache. Both improved with a snack and rest, and he returned to the inpatient unit for the night. The tremor continued to wax and wane throughout the evening, often severe enough to prevent the use of his right hand. After he fell asleep lying on his right hand, he was awakened by the tremor and was then sent for evaluation in the emergency department (ED) of an adjacent academic hospital, where the neurologists thought it unlikely to be a focal seizure but also found it atypical for a tremor. The tremor had receded at about 3 AM, and he was discharged from the ED without further workup or firm diagnosis at 6 AM. He was discharged from the iTBS treatment phase of the study and moved into the follow-up phase.

**Figure 2:**
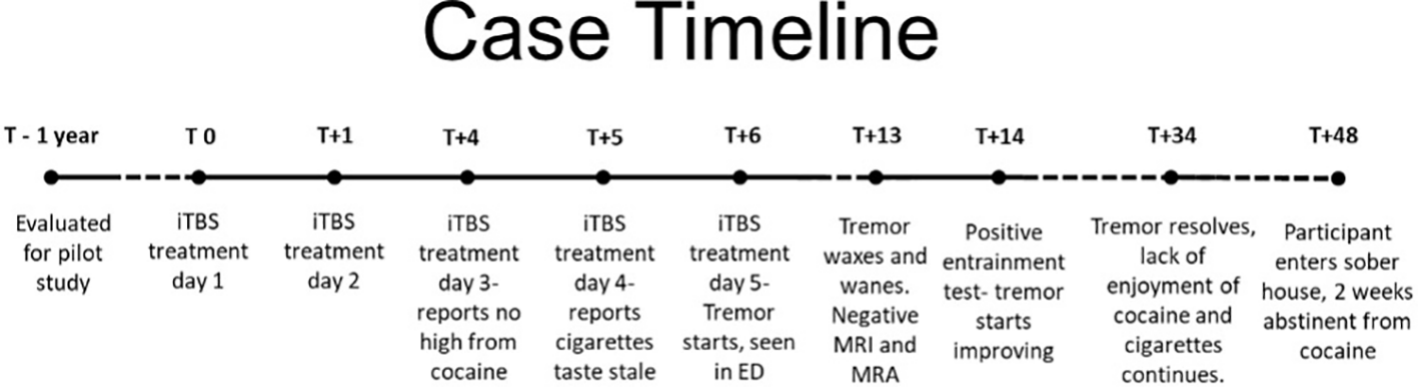
Progression of participant participation in the study relative to their first iTBS treatment day (T0). Tremor first appeared and was evaluated 6 days after the first iTBS treatment day (T+6).

Over the next week, the tremor continued to wax and wane, often causing difficulty with functioning and sometimes being said to be waking him from sleep. Further clinical workup was pursued, which included an MRI with contrast and a magnetic resonance angiography (MRA; both unremarkable), and an EEG was ordered but could not be completed before the tremor had resolved after 28 days, study day 34 (see **Figure 2**). Video of the tremor along with medical records were reviewed by three outside consultants, two neurologists, and one psychiatrist. Opinions ranged from likely to be an organic tremor but of unclear etiology to likely a functional tremor. One of the neurology consultants recommended an entrainment test, which was conducted by BJS a week after the onset of the tremor. It revealed clear signs of a functional tremor with a reduced tremor in the affected (right) hand during a patterned movement of the unaffected (left) hand and syncing of the residual right-hand movement with the purposeful movement of the left hand. On seeing the tremor change with left-hand patterned movements, the participant remarked, “Maybe it’s a behavioral thing!” He was advised to do exercises with his left hand, such as bouncing a ball against a wall or practicing piano (one of his hobbies), to help reduce the tremor.

The blind was broken 8 days after tremors started, study day 14, revealing the participant had received sham iTBS. Only the study physician (BJS) was unblinded as the participant continued through follow-up visits with VRS.

His tremors continued, although markedly reduced and no longer interfered with his functioning, for about 2 weeks before resolving completely, about a month after they started. He was seen three times per week in the CUD contingency management phase of the study over the next 6 weeks, except for a 10-day stretch during which his mother suffered a severe stroke and died, and he underwent an urgent, minor urologic procedure. He used cocaine four more times and continued to report he did not get any high from his use. He continued to report his cigarettes tasted stale and reduced his use drastically, going a week without any cigarettes before resuming one-half to 1 cigarette with coworkers about every other day, down from a baseline of 10–20 cigarettes per day. He smoked cannabis twice during this period, once when he found a forgotten joint in a coat pocket and once with a friend. Although he reported getting high from the cannabis, he did not resume daily usage. He also continued to report vivid dreams with nondrug content. He complained that his normal coping mechanisms (cocaine and cigarettes) were no longer effective, and he was working with his therapist to develop new ways to cope with stress. He was accepted into a sober house program and was lost to follow-up about 6 weeks after iTBS was halted. He was 2 weeks abstinent from cocaine at that time and 2 weeks tremor-free when he stopped responding to repeated contact attempts (**Figure 2**).

## Discussion

We report a case of functional tremor, persisting for about 4 weeks, that developed in a participant assigned to the sham arm of accelerated iTBS treatment for CUD (NCT02927236). Notably, this participant experienced a strong clinical response [similar to reports from our participants who received open-label, active iTBS (6)] with a loss of ability to get high from cocaine or enjoy cigarette smoking as he had prior to sham iTBS, with markedly reduced craving for and use of all drugs. Also, he reported a transformation of his vivid drug dreams into equally vivid nondrug dreams. The fact that this plethora of clinical changes occurred despite having received sham iTBS stimulation is remarkable.

rTMS has several features that may induce an enhanced placebo effect. These include lengthy interaction with a team delivering the treatment that involves numerous hands-on procedures with complex machinery, painful somatic stimulation induced by either the active rTMS and/or the surface forehead electrodes, electrical and visual monitoring of MEPs, and use of the participant’s structural brain scan for rTMS targeting (11). This participant had also waited about one year to participate in the study and had particularly high expectations for treatment benefits. rTMS has been known to produce dramatic placebo responses (2, 3), and it is thus likely that placebo effects account for the current presentation. A placebo effect could explain the clinical improvements based on the belief of expectations of benefit (2, 12). The development of a functional tremor may have a similar pathophysiology based in this situation on belief of illness (13). It is well known that functional symptoms can be produced and improved with a placebo (14). This circumstance suggests the interesting possibility that the development of a functional disorder could be a marker of a placebo response.

However, another possibility comes from the literature on low-field rTMS, including electrical fields generated by the sham electrodes used in the current study. Contrary to the assumption in much rTMS work that stimulation must cause cortical action potentials to be effective, there is mounting evidence that far weaker stimulation can still have biological effects, in some cases very similar to suprathreshold stimulation (4, 5). These observations suggest that the weak electrical fields of placebo rTMS may produce biological effects that could account for the clinical improvement and functional tremor in this participant.

This case draws attention to the importance of the sham condition in rTMS research, as has been pointed out previously (3, 11, 15). The problem of adequately controlling for the nonspecific effects of rTMS is further complicated by the possibility that sham systems used to mimic the sensory features of active rTMS may have biological effects themselves. This issue requires further attention as various forms of rTMS are developed for therapeutic purposes across clinical populations.

## Data availability statement

The raw data supporting the conclusions of this article will be made available by the authors, without undue reservation.

## Ethics statement

The studies involving humans were approved by National Institute on Drug Abuse Institutional Review Board and the Food and Drug Administration. The studies were conducted in accordance with the local legislation and institutional requirements. The participants provided their written informed consent to participate in this study. Written informed consent was obtained from the individual(s) for the publication of any potentially identifiable images or data included in this article.

## Author contributions

VS: Conceptualization, Data curation, Formal analysis, Funding acquisition, Methodology, Project administration, Supervision, Validation, Writing – original draft, Writing – review & editing. AR: Writing – original draft, Writing – review & editing. NP: Writing – original draft, Writing – review & editing. MH: Writing – original draft, Writing – review & editing. ES: Conceptualization, Funding acquisition, Methodology, Resources, Supervision, Writing – original draft, Writing – review & editing. BS: Conceptualization, Investigation, Methodology, Project administration, Supervision, Writing – original draft, Writing – review & editing.
